# Predictors of Medication Adherence and Blood Pressure Control among Saudi Hypertensive Patients Attending Primary Care Clinics: A Cross-Sectional Study

**DOI:** 10.1371/journal.pone.0171255

**Published:** 2017-01-30

**Authors:** Sarah M. Khayyat, Salwa M. Saeed Khayyat, Raghda S. Hyat Alhazmi, Mahmoud M. A. Mohamed, Muhammad Abdul Hadi

**Affiliations:** 1 Department of Clinical Pharmacy, Faculty of Pharmacy, Umm Al-Qura University, Makkah, Saudi Arabia; 2 Public Health Centers, Ministry of Health, Makkah, Saudi Arabia; 3 Pharmaceutical Research Center, Deanship of Scientific Research, Umm Al-Qura University, Makkah, Saudi Arabia; 4 School of Healthcare, University of Leeds, Leeds, United Kingdom; Florida International University Herbert Wertheim College of Medicine, UNITED STATES

## Abstract

**Purpose:**

To assess the level of medication adherence and to investigate predictors of medication adherence and blood pressure control among hypertensive patients attending primary healthcare clinics in Makkah, Saudi Arabia.

**Patients and methods:**

Hypertensive patients meeting the eligibility criteria were recruited from eight primary care clinics between January and May 2016 for this study. The patients completed Arabic version of Morisky Medication Adherence Scale (MMAS-8), an eight-item validated, self-reported measure to assess medication adherence. A structured data collection form was used to record patients’ sociodemographic, medical and medication data.

**Results:**

Two hundred and four patients, of which 71.6% were females, participated in the study. Patients’ mean age was 59.1 (SD 12.2). The mean number of medication used by patients was 4.4 (SD 1.89). More than half (110; 54%) of the patients were non-adherent to their medications (MMAS score < 6). Binary regression analysis showed that highly adherent patients (MMAS score = 8) were about five times (OR 4.91 [95%CI: 1.85–12.93; P = 0.01]) more likely to have controlled blood pressure compared to low adherent patients. Female gender (OR 0.40 [95% CI: 0.20–0.80; P = 0.01]), Age > 65 years (OR 2.0 [95% CI: 1.0–4.2; P = 0.04]), and being diabetic (OR 0.25 [95% CI: 0.1–0.6; P = 0.04]) were found to be independent predictors of medication adherence.

**Conclusion:**

Medication adherence is alarmingly low among hypertensive patients attending primary care clinics in Saudi Arabia which may partly explain observed poor blood pressure control. There is a clear need to educate patients about the importance of medication adherence and its impact on improving clinical outcomes. Future research should identify barriers to medication adherence among Saudi hypertensive patients.

## Introduction

Globally, hypertension is a serious public health problem as it is one of the leading preventable causes of morbidity and mortality [1, 2]. As of 2008, the World Health Organization (WHO) reported that hypertension affected 1 billion patients across the globe, 40% of adults aged 25 years and above. Hypertension accounts for 9.4 million deaths every year either due to heart diseases (45%) or stroke (51%) worldwide [1]. Given the high humanistic and economic cost associated with hypertension, early detection, proper management and control of blood pressure is crucial to avoid long term complications of hypertension [2].

Pharmacotherapy together with lifestyle modifications remain the cornerstone in the management of hypertension [3, 4]. Medication adherence is the key in achieving the desired clinical outcomes [5]. The WHO defines medication adherence as “the extent to which a person's behavior—taking medication, following a diet, and/or executing lifestyle changes, corresponds with agreed recommendations from a health care provider” [5]. A number of studies conducted internationally have reported significant association between medication adherence and blood pressure control [6–12]. Poor medication adherence is associated with various medical/psychosocial complications, poorer health-related quality of life and increased the health care costs [5, 13, 14].

In Saudi Arabia, hypertension has been estimated to be the leading risk factor for death [15]. It has been estimated that about one in four adults (age 15–64 years) have hypertension [16]. Poor blood pressure control among Saudi patients is well documented [16,17]. A large national study reported that 63% (total N = 1213) of the hypertensive patients had uncontrolled blood pressure, an alarmingly high rate [16]. However, there is scarcity of data regarding Saudi patients’ adherence to antihypertensive medications, especially within primary care setting [18–20]. Since hypertension is primarily managed within primary care, it is important to assess the level of medication adherence and factors affecting adherence, so that necessary measures can be taken within the primary care settings in order to improve patients’ adherence to their medications, prevent long-term negative consequences of non-adherence and reduce burden on secondary care.

The main objectives of the current study were to: (1) assess the level of medication adherence in adult hypertensive patients attending primary care clinics; (2) identify socio-demographic and clinical characteristics that affect patients’ adherence and blood pressure control.

## Material and Methods

### Ethical approval

The study was approved by the local Institutional Review Board (IRB) at the Faculty of Pharmacy, Umm Al-Qura University, Makkah, Saudi Arabia. In addition, ethics and governance approval were also obtained from the General Directorate of Health Affairs Makkah Region, Ministry of Health, Saudi Arabia (Ref # M/47/402/2334855). Each participant completed a written consent form before enrollment.

### Participants and settings

This was a prospective cross-sectional study conducted between January and May 2016. Patients’ data were collected from eight different primary healthcare clinics (PHC) in Makkah city. Convenient sampling technique was used to recruit these clinics. Patients meeting the following inclusion and exclusion criteria were included: confirmed diagnosis of hypertension for more than 6 months; Age > 18 years; taking at least one antihypertensive medication; and ability to communicate in Arabic. Pregnant women, patients with mental health issues and dementia were excluded from the study. The attending general physician (GP) screened all patients during the study period and assessed for eligibility.

A universal sampling techniques was used to recruit the patients. All patients meeting inclusion and exclusion criteria were requested to participate in the study and were asked to complete a written consent form. For the purpose of this study, the goal of controlled blood pressure (BP) was defined in accordance to the NICE guideline (2011) for hypertension in adult patients [2]. Patients’ blood pressure was considered controlled if: (1) Patients under 80 years old with treated hypertension have BP under 140/90 mmHg. (2) Patients aged 80 years or over with treated hypertension have BP under 150/90 mmHg. (3) Patients with both hypertension and diabetes mellitus have BP less than 140/80 mmHg or less than 130/80 mmHg (in presence of any kidney, eye or cerebrovascular damage).

### Data collection

For assessing medication adherence, patients were requested to complete a structured and validated 8-item questionnaire, Morisky Medication Adherence Scale (MMAS-8) [21–23]. Since Arabic is the national language of Saudi Arabia, a validated Arabic translated version of MMAS-8 was used in this study. MMAS-8 has been widely used for assessing patients’ adherence to their medications [6–8, 24–27].

The first seven items of MMAS-8 have dichotomous responses (Yes/No) to avoid acquiescence bias, whereas the eighth item has 5-point Likert scale response indicating low to high level of adherence [21–23]. Total summated adherence score range between 0 and 8. Using the standard scoring criteria, a score less than 6 was considered low adherence, between 6 and less than 8 as medium adherence and 8 as high adherence. A license agreement was signed and permission was obtained from appropriate authority to use MMAS-8 in this study [21–23].

A standardized, structured data collection form was used to gather patients’ sociodemographic, medical and medication data. The respective attending physician completed this form by reviewing patients’ medical record.

### Statistical analysis

Statistical analyses were conducted by SPSS software (Version 23.0. Armonk, NY: IBM Corp). All statistical tests used were two-tailed. The alpha level of significance for all statistical tests was 0.05 unless otherwise specified. Binary logistic regression analysis using the backward stepwise likelihood-ratio method was conducted to determine factors that could significantly predict adherence as well as blood pressure control. Correlation and Hosmer-Lemeshow Goodness-of-Fit Tests were done to select best prediction model.

## Results

### Demographics and health status

A total of 204 patients participated in the study. More than half of the sampled patients (71.6%) were females with an overall mean age of 59.1 (SD = ± 12.21). The majority of the study population was Saudis (93.1%), married (76%), and literate (48%). Most of the patients 132 (64.7%) were obese (BMI; ≥ 30 kg/m2) and only 15 patients (7.4%) had normal body mass index (BMI; 18.5 to 24.9 kg/m2). Of the 204 hypertensive patients sampled, 146 (71.6%) had concomitant diabetes and 93 (45.6%) had hyperlipidemia (Table 1).

**Table 1:** Patients’ demographics and health status.

Demographic variables	Total study population (N = 204)	
	N	%
**Gender**		
Female	146	71.6
Male	58	28.4
**Age**	Mean (SD) 59.1 (12.2)	
19–35	5	2.5
36–50	47	23
51–65	103	50.5
66–85	41	20.1
>85	8	3.9
**BMI**		
Normal	15	7.4
Overweight	57	27.9
Obese	132	64.7
**Level of Education**		
Illiterate	98	48
Elementary	46	22.5
High school	35	17.2
BS degree or higher	25	12.3
**Nationality**		
Saudi	190	93.1
Non-Saudi	14	6.9
**Employment status**		
Employed	33	16.2
Unemployed	130	63.7
Retired	41	20.1
**Income status**		
Satisfied	160	78.4
Unsatisfied	44	21.6
**Marital status**		
Single	17	8.3
Married	14	6.9
Divorced	95	46.6
Widowed	78	38.2
**Number of children**		
No children	17	8.3
1–2	14	6.9
3–5	95	46.6
> 5	78	38.2
**Smoking status**		
Smoking	25	12.3
Non-smoking	179	87.7
**Number of comorbidities**		
≤ 2	98	48
3	93	45.6
≥ 4	13	6.4
**Specific comorbidity**		
Patients with DM	146	71.6
Patients with HF	2	1
Patient with Hyperlipidemia	93	45.6
**Number of current medications**	Mean 4.4 (1.89)	
1	10	4.9
2	25	12.3
3	34	16.7
4	48	23.5
5	34	16.7
≥6	53	25.9
**Blood Pressure**		
Controlled	142	69.6
Uncontrolled	62	30.4

**Abbreviations:** N, number of patients; SD, standard deviation; BMI, Body mass index; DM, diabetes mellitus; HF, heart failure.

### Adherence rate

Adherence scores ranged from 0 to 8 on MMAS-8. Based on the MMAS-8 score, patients were categorized into three groups as described in the methods section: low adherence (MMAS-8 score < 6), medium adherence (MMAS-8 score 6 to < 8) or high adherence (MMAS-8 score 8). The frequency distribution is shown in Table 2. More than half of the respondents (54%) had low adherence, while 23.5% and 22.5% had medium and high adherence level respectively. Responses for each of the MMAS-8 are summarized in Table 3.

**Table 2:** Adherence level among hypertensive patients stratified by blood pressure control.

Adherence level (score)	Blood pressure	Total study population (N = 204)		
		N	%	Total (%)
Low adherence (< 6)	Controlled	67	32.8	110 (54)
	Uncontrolled	43	21.1	
Medium adherence (6 to <8)	Controlled	35	17.2	48 (23.5)
	Uncontrolled	13	6.4	
High adherence (= 8)	Controlled	40	19.6	46 (22.5)
	Uncontrolled	6	2.9	

**Abbreviations:** N, number of patients.

**Table 3:** Responses for each question in the (MMAS-8) scale.

Questions			Total study population (N = 204)		
			Yes (%)		No (%)
#1			90 (44.1)		114 (55.9)
#2			62 (30.4)		142 (69.6)
#3			49 (24)		155 (76)
#4			61 (29.9)		143 (70.1)
#5			18 (8.8)		186 (91.2)
#6			37 (18.1)		167 (81.9)
#7			111 (54.4)		93 (45.6)
#8	All the time	Usually	Sometimes	Once in a while	Never/ Rarely
	0 (0)	1 (0.5)	50 (24.5)	63 (30.9)	90 (44.1)

**Abbreviations:** MMAS-8, Morisky Medication Adherence Scale (8-item); N, number of patients. **Notes:** Use of the MMAS is protected by US copyright laws. Permission for use is required. A license agreement is available from: Donald E. Morisky, ScD, ScM, MSPH, Professor, Department of Community Health Sciences, UCLA Fielding School of Public Health, 650 Charles E. Young Drive South, Los Angeles, CA 90095–1772, dmorisky@ucla.edu. The scale’s questions are available in the originally published article [21].

For the purpose of analysis, patients were classified in to two (adherent and non-adherent) rather than three categories (low, medium, high) based on MMAS-8 scores: non-adherent (MMAS-8 score < 6) and adherent (MMAS-8 score ≥ 6).

Table 4 shows results of the binary logistic regression analysis identifying the factors predicting medication adherence. The model can explain 14% of the change in the adherence level (P = 0.004). The odds of adherence for female patients are 60% less than the male patients (OR 0.40 [95% CI: 0.2–0.8; P = 0.01]). Patients aged > 65 years have twice the odds of medication adherence compared to patients less than 65 years old (OR 2.0 [95% CI: 1.0–4.2; P = 0.04]). Non-diabetic patients are 74% less likely to be adherent compared to diabetic patients (OR 0.2 [95% CI: 0.1–0.6; P = 0.04]).

**Table 4:** Binary logistic regression analysis for factors predicting medication adherence.

Parameter	Non-Adherent N (%)	Adherent N (%)	OR	95% CI for OR Lower—Upper	P-value
**Marital status**					
Widow	22 (69)	10 (31)	1		
Single	2 (40)	3 (60)	2.55	0.3–19.8	0.36
Married	79 (51)	76 (49)	2.74	1.0–7.0	0.03*
Divorced	7 (58)	5 (42)	2.16	0.5–9.1	0.29
**Diabetes**					
Diabetic	86 (59)	60 (41)	1		
Non-diabetic	24 (41)	34 (59)	0.26	0.1–0.6	0.04*
**Gender**					
Male	23 (40)	35 (60)	1		
Female	87 (59)	59 (41)	0.40	0.2–0.8	0.01*
**Age**					
≤ 65	89 (57)	66 (43)	1		
> 65	21 (43)	28 (57)	2.12	1.0–4.2	0.04*
**Number of comorbidities**					
≤3	84 (53)	76 (47)	1		
>3	26 (59)	18 (41)	1.11	0.3–3.6	0.91
**Number of medications**					
>6	36 (52)	33 (48)	1		
4–6	55 (53)	48 (47)	1.22	0.2–5.4	0.78
≤3	19 (59)	13 (41)	1.11	0.1–11.9	0.89

**Notes:** x^2^ = 22.65; df = 7; N = 204; P = 0.004; R^2^ = 0.139. **Abbreviations:** N, number of patients; CI, Confidence Interval; OR, Odds ratio; df, degrees of freedom.

* indicates statistically significant results.

### Blood pressure control

Blood pressure control was better among patients with high level of adherence than those with low adherence rate. Table 5 summarizes the predictors of blood pressure control using the binary logistic regression. About 13.6% of the change in blood pressure can be explained by this model (P = 0.002). The results showed that the odds of blood pressure control for overweight patients was 53% less than normal weight patient, (OR 0.4 [95% CI: 0.2–0.9; P = 0.03]) after adjusting for gender, age, and level of adherence. In addition, patients highly adherent to their medications are five times more likely to have controlled blood pressure compared to low adherent patients. (OR 4.9 [95% CI: 1.8–12.9; P = 0.001]) after adjusting for other confounders.

**Table 5:** Binary logistic regression analysis identifying factors predicting BP control.

Predictor variables	Blood pressure N (%)		OR	95% CI for OR Lower—Upper		P-value
	Controlled	Uncontrolled				
**Gender**						
Male	41(71)	17 (29)	1			
Female	101 (69)	45 (31)	1.21	0.5	2.4	0.59
**Age**						
≤ 65	112 (72)	43 (28)	1			
> 65	30 (61)	19 (39)	0.50	0.2	1.0	0.05
**BMI**						
Normal weight	8 (5.6)	7 (11.3)	1			
Overweight	35 (24.6)	22 (35.5)	0.47	0.2	0.9	0.03*
Obese	99 (69.7)	33 (53.2)	0.31	0.09	1.0	0.05
**Level of adherence**						
Low adherence	67 (61)	43 (39)	1			
Medium adherence	35 (73)	13 (27)	1.96	0.9	4.2	0.10
High adherence	40 (87)	6 (13)	4.90	1.8	12.9	0.001*

**Notes:** x^2^ = 20.590; df = 6; N = 204; P = 0.002; R^2^ = 0.136; Odds ratios are non-standardized. **Abbreviations:** BP, blood pressure; n, number of patients; CI, confidence interval; OR, odds ratio; df, degrees of freedom; N, number of patients.

*donates statistically significance.

## Discussion

The aim of current study was to assess the extent of medication adherence among adult hypertensive patients in the primary care setting. Identifying patient groups which are likely to have poor blood pressure control is crucial for both clinicians and health policy makers so that appropriate interventions targeting specifically these patient groups can be designed and implemented. The present study was designed to build on earlier studies which have documented poor blood pressure control among Saudi patients by identifying factors affecting blood pressure control and medication adherence [18–20, 24].

The current study found that the majority of the sampled hypertensive patients had low levels of medication adherence. Poor adherence to antihypertensive medications is not only associated with poor blood pressure control but also accelerates development of hypertension related complications and increases hospital admissions rate [5,13,14]. In line with the findings of our study, another local study conducted in Riyadh, Saudi Arabia among patients with long term conditions also reported low levels of medication adherence among patients [24]. Various international studies [6, 7, 25, 27], have also documented similar poor adherence rates which is concerning given the negative consequences associated with non-adherence.

The overall percentage of adherent patients in current study was less than the adherence rate previously reported in Saudi Arabia. Previous studies have estimated adherence rates between 35% and 53% [18–20]. The difference in the percentages of adherence rate between the literature and current study may be related to the difference in the study population, patients’ knowledge, health literacy, and complexity of patients’ regimens and health conditions.

In the present study, gender, age and history of diabetes were found to be independent predictors associated with higher medication adherence rates. A number of local and international studies have studied gender differences in relation to medication adherence and reported inconsistent results [18–20, 24, 27]. Low levels of medication adherence among female patients have been reported in this study and in a couple of other studies conducted in Saudi Arabia [18, 20]. It has been documented that women with long term conditions are less likely to receive medical treatment and monitoring as recommended by clinical guidelines [28]. Lack of adequate monitoring may partly explain low levels of medication adherence among females.

Patients’ age was positively associated with the adherence score in this study and other international studies [6, 19, 24, 26, 27]. For instance, one of the studies which was conducted in Saudi Arabia, reported better adherence rate among hypertensive aged over 55 years than those younger than 55 years (48.5% versus 26.2%, P < 0.001) [19]. Higher medication adherence among older patients can be explained by the presence of a caregiver who would help them in taking their medications. Furthermore, middle aged patients usually have work related commitments and other priorities in their lives, therefore may not be able to attend their clinic appointments and take their medicines as prescribed [29].

In line with the findings of previous studies [6–8, 11, 12, 18–21], medication adherence was found to be an independent predictor of blood pressure control. In literature, a number of factors affecting medication adherence, and subsequently therapeutic outcomes, have been identified and are classified into: patient-related factors (e.g. socio-demographic factors, individual’s knowledge and skills, individual’s beliefs and perceptions, and physical/mental ability), health system-related factors (e.g. quality of healthcare services, cost of treatment and patient resources), and provider-related factors (e.g. provider-patient relationship and communication) [5,30].

Another predictor of blood pressure control found in the present study was BMI, with normal weight patients had better BP control compared to overweight patients. Several studies have reported positive impact of lifestyle modifications such as weight reduction, healthful dietary plan, regular physical activity and other behavioral changes on not only reducing systolic and diastolic blood pressure but also on preventing complications associated with hypertension [4,5,17,31]. Therefore, physicians should educate patients about the benefits of healthy life style and encourage lifestyle modifications, if required, especially weight reduction and smoking cessation.

Mixed results have been reported regarding the association between number of medications and adherence level [6–8, 19, 24, 27, 32, 33]. In another study conducted in Saudi Arabia, patients with chronic diseases, multiple medications and complex regimens were more likely to adhere to their long-term medications [24]. However, the present study could not find any association between number of medications and adherence level. The impact of poor adherence rate on developing of hypertension-related complications was out of the scope of the present study. However, one Saudi study has reported a positive impact of adherence on preventing hypertension-related complications [18]. The study presented significantly less complications among patients with fair-to-good compliance compared to those with poor compliance [18].

### Implications for clinical practice

The study findings have significant implications for controlling BP in primary care settings in Saudi Arabia. A clear need to design and implement interventions to improve adherence among hypertensive patients has been recognized. Furthermore, specific groups of patients which are likely to be non-adherent (females, patients age < 65 years, widows) have also been identified. Improving patients’ poor adherence to the antihypertensive medications could be achieved by improving their knowledge, motivation, skills and resources to follow physicians and/or other healthcare providers’ recommendations [7, 8, 18, 25, 26]. For instance, some of the supporting interventions to increase patients’ adherence include: advising the patient to record his/her medicine-taking, encouraging the patient to monitor his/her blood pressure and other hypertension-related complications, simplifying the dosing regimen by the healthcare providers, and finally, providing annual review of care to the patient could help in improving the adherence rate and the control of his/her blood pressure [2].

There are some limitations of current study. Firstly, the use of self-reported questionnaires may under or over-reported the true incidence of patients’ poor adherence. Furthermore, questionnaires are inherent to recall bias. There are a number of methods of assessing medication adherence (patient interviews, diaries, questionnaires, pill count, and pharmacy refills and claims data) and no single method is perfect [34]. Therefore, triangulation of two methods is recommended, if possible [34]. MMAS-8 was used in the present study as it is one the most widely used self-reported measure to assesses medication adherence which allowed the authors to compare their findings with the findings of other studies both locally and internationally. Secondly, the study didn’t take into consideration the chronicity of hypertension and consider it as one of the confounders. In addition, impact of other patient-related factors such as: patients’ knowledge and background, their beliefs and perception about their disease and treatment, and their physical/mental ability should be evaluated in future research [30]. This will be an important area for future research because health behavior researchers have provided evidence that patients’ ideas about their diseases and medications is one of the predictors of medication adherence [25, 26, 30].

## Conclusion

Adherence to medications is alarmingly low among hypertensive patients attending primary care clinics in Makkah, Saudi Arabia. This calls for a well-designed educational intervention especially targeting high risk groups (females, patients’ age < 65 years and widows). Since non-adherence has been associated with increased hospitalizations and hypertension-related complications, it is important to educate patients about the importance of medication adherence. Barriers to medication adherence should also be explored among patients attending primary care clinics in Saudi Arabia. Without optimizing the use of medicines through patient-professional partnership, it will be unlikely to achieve desired clinical outcomes for Saudi patients.
