# Correction: Predictors of Medication Adherence and Blood Pressure Control among Saudi Hypertensive Patients Attending Primary Care Clinics: A Cross-Sectional Study

**DOI:** 10.1371/journal.pone.0187614

**Published:** 2017-10-31

**Authors:** The PLOS ONE Staff

## Notice of Republication

Table 3 was included in the article without full permission. The table was removed from the HTML and PDF versions of this article on July 21, 2017. Please download this article again to view the correct version. Readers can access the MMAS-8 scale through the original publication [2].
